# CircRNA CTNNB1 (circCTNNB1) ameliorates cerebral ischemia/reperfusion injury by sponging miR-96-5p to up-regulate scavenger receptor class B type 1 (SRB1) expression

**DOI:** 10.1080/21655979.2022.2061304

**Published:** 2022-04-17

**Authors:** Chun Chen, Xiaolong Chang, Shifei Zhang, Qi Zhao, Chunyan Lei

**Affiliations:** Department of Neurology, First Affiliated Hospital of Kunming Medical University, Kunming, China

**Keywords:** Cerebral ischemia-reperfusion injury, oxygen glucose deprivation and reperfusion, blood-brain barrier, circRNA CTNNB1, scavenger receptor class B type I, miR-96-5p

## Abstract

Emerging studies show that circRNA catenin beta 1 (circCTNNB1) plays a critical role in cancer. However, the expression and function of circCTNNB1 in cerebral ischemia/reperfusion injury (IRI) have not been reported. The present study discovered that circCTNNB1 and scavenger receptor class B type 1 (SRB1) expression levels were significantly down-regulated in mouse astrocytes (mAS) treated with oxygen glucose deprivation and reperfusion (OGD/R), and similar results were observed in a mouse middle cerebral artery occlusion model. Overexpression of circCTNNB1 alleviated cell apoptosis, oxidative stress and the inflammatory response induced by OGD/R *in vitro*. Up-regulation of circCTNNB1 increased SRB1 expression levels to protect mAS cells from OGD/R-induced damage. CircCTNNB1 and SRB1 interacted with miR-96-5p, and the overexpression of miR-96-5p efficiently reversed the function of circCTNNB1 in OGD/R-treated mAS cells. CircCTNNB1 protected against cerebral ischemia-reperfusion injury by up-regulating SRB1 *in vivo*. In conclusion, our findings suggest that circCTNNB1 acts as a competitive endogenous RNA for miR-96-5p to alleviate cerebral IRI, which provides novel evidence that circCTNNB1 and SRB1 may be biomarkers and therapeutic targets for cerebral IRI.

## Graphical abstract

**Figure:**
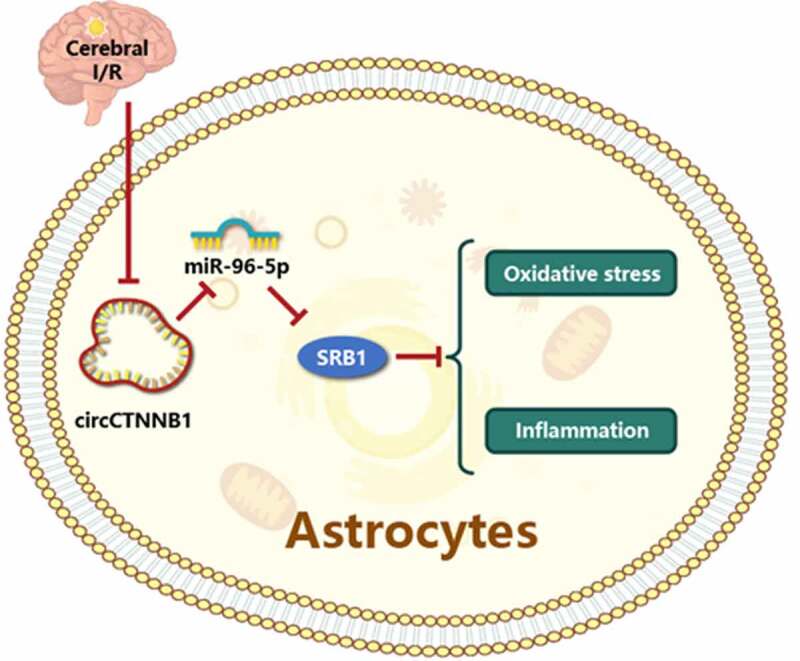

## Introduction

Cerebral ischemia-reperfusion injury (IRI) is a normal clinical pathogenic process that may produce major brain malfunction, which results in extremely high mortality and disability [1]. Ischemia reperfusion induces apoptosis, the overproduction of reactive oxygen species (ROS), and recruitment of inflammatory cells, which lead to ischemic brain damage [2]. Inflammatory events occurring at the blood-brain barrier (BBB) throughout cerebral ischemia are critical for the pathogenesis of tissue damage in IRI [3]. The BBB is a unique part of the central nervous system. This structure includes astrocytes, pericytes, neighboring neurons and capillaries. BBB malfunction starts with the degradation of tight junction proteins and the opening of barrier junctions as a result of oxidative stress caused by the production of free radicals following ischemia [4]. Aquaporin-4 increases, and brain edema exacerbates irreversible brain damage [5]. However, the molecular mechanism of cerebral IRI is not clear, which leads to a lack of effective therapy [6]. Therefore, it is important to discover a novel and effective treatment strategy to treat cerebral IRI.

Recent research on high-throughput RNA sequencing has determined that only 2% of whole genome transcripts were protein-coding RNAs and 98% were non-coding RNAs [7]. Various non-coding RNAs were discovered, including long non-coding RNAs, circular RNAs (circRNAs), microRNAs (miRNAs), small interfering RNAs, and small nuclear RNAs [8]. The structure of circRNAs is different from other non-coding RNAs because the 3’ head and 5’ end covalently combine to form a closed loop structure [9]. Due to their specific structure, uncertain function, and low abundance, circRNAs were considered genomic junk for decades [10]. However, accumulating evidence indicates that circRNAs participate in gene regulation in multiple ways [11]. More research has been performed on circRNAs and demonstrated binding with the miRNA response element, which served as a competitive endogenous RNA of the miRNA binding site and regulated the activity of miRNAs [12]. circCTNNB1 is a circRNA involved in tumor growth, invasion and metastasis [13]. Notably, the protein encoding gene CTNNB1 (β-catenin) is a homolog of circCTNNB1 and plays a key role in maintaining the blood-brain barrier and the dynamic balance of the central nervous system [14,15]. However, the function of circCTNNB1 in maintaining BBB integrity is poorly understood.

High-density lipoprotein (HDL) has protective effects on cardio-cerebral blood vessels, such as anti-atherosclerosis and reducing BBB damage from stroke [16,17]., HDL plays numerous roles in vivo via a series of signaling pathways, but the molecular mechanisms are not fully understood. Scavenger receptor class B member 1 (SRB1) is an HDL receptor that mediates the bidirectional flow of cholesterol and lipids between HDL and cells [18,19]. Several studies showed that its biological functions included, but were not limited to, anti-septicemia, control of inducible steroid production to protect the liver, protecting immune dysfunction, and promoting bone differentiation [20–22]. Although SRB1 plays an active role in many diseases, its effects on neurological diseases have not been investigated.

Based on the aforementioned data and findings, we hypothesized that circCTNNB1 would maintain BBB integrity to ameliorate cerebral IRI via modulation of SRB1 expression. To validate our hypothesis, we established a middle cerebral artery occlusion (MCAO) model and an astrocyte oxygen glucose deprivation and reperfusion (OGD/R) model to examine the role of circCTNNB1 and its downstream mechanism in cerebral IRI. We revealed the roles of core circCTNNB in cerebral IRI, which contribute to early targeted therapy for patients.

## Materials and methods

### Cell culture and OGD/R model

As previously described, primary mouse astrocytes (mAS) were extracted from post‐natal day 7 cerebral cortexes [23]. Brain regions were dissected, mechanically dissociated and incubated with trypsin before trituration, washing and filtering. Cells were counted, plated at a density of 10^7^ cells in 75 cm^2^ tissue culture flasks pre‐coated with poly‐D‐lysine and grown at 37°C in 5% CO_2_ in Dulbecco’s modified Eagle’s medium (Solarbio; Beijing, China) containing 10% fetal bovine serum (Solarbio), 100 U/mL penicillin (Solarbio) and 100 µg/mL streptomycin (Solarbio). Purified mAS cells were stained with anti-ACSC2-APC (Miltenyi Biotec, Germany) and subjected to flow cytometry analysis on a FACS-Canto flow cytometer (Becton Dickinson; San Jose, USA).

The mAS cells were subjected to OGD to simulate an *in vitro* ischemic‐like condition as previously described [24]. The culture medium was replaced with deoxygenated glucose‐free DMEM, and the cells were incubated for 6 hours in a hypoxic chamber containing 5% CO_2_, 1% O_2_ and 94% N_2_. The mAS cells were reoxygenated in glucose‐containing DMEM under normal culture conditions for 1, 24 and 48 hours for reoxygenation. The overall treatment protocol is referred to as OGD/R treatment.

### Transfection

Overexpression (OE)-circCTNNB1, OE-empty vector (EV), siRNA (si)-SRB1, si-EV, miR-96-5p mimic or negative control (NC) mimic, or two of these treatments were transfected into cells using Lipofectamine™ 3000 Transfection reagent (Invitrogen; Carlsbad, USA) at 37°C. After 6 hours of transfection, mAS cells underwent OGD/R treatment (6‐hour OGD and 24‐hour reoxygenation). The transfection efficiencies of OE-circCTNNB1 and miR-96-5p mimic were detected using reverse transcription‑quantitative PCR (RT-qPCR). The transfection efficiency of si-SRB1 was detected using Western blotting.

### RT-qPCR

To determine circCTNNB1 and miR-96-5p levels, 5 × 10^6^ mAS cells per sample were harvested following treatment or transfection. Total RNA was extracted using TRIzol Reagent (Invitrogen). The expression levels of circCTNNB1 and miR-96-5p were measured using a QuantiNova SYBR Green PCR Kit (TaKaRa; Tokyo, Japan). The following specific primers were used: circCTNNB1, forward, 5′-AAGGACACAAAAACTCTCTTCTTCC-3′ and reverse, 5′-AGAGGGGGCATGTAAAAGAAAA-3′; miR-96-5p, forward, 5′-TTTGGCACTAGCACAT-3′ and reverse, 5′-GAGCAGGCTGGAGAA-3′; GAPHD, forward, 5′-AGAAGGCTGGGGCTCATTTG-3′, reverse, 5′-AGGGGCCATCCACAGTCTTC-3′; U6, forward, 5′-AACGAGACGACGACAGAC-3′ and reverse, 5′-GCAAATTCGTGAAGCGTTCCATA-3′. The PCR experiments were performed in a LightCycler 480 (Roche; Basel, Switzerland). Amplification was performed at 95°C for 10s for 40 cycles (at 95°C for 10s; at 60°C for 30s). The relative expression level was calculated using the 2^−ΔΔCt^ method [25] and normalized to GAPDH (mRNA) and U6 (miRNA) levels.

### Western blotting

Proteins were extracted using protein lysis solution (Solarbio) according to the manufacturer’s protocol. The purity of the protein in the extracts was examined using the bicinchoninic acid method. Electrophoresis using a 10% SDS-polyacrylamide gel was used to separate the samples, which were transferred to a PVDF membrane. Nonspecific protein binding was inhibited by the addition of blocking solution (5% milk, 20 mM Tris-HCl [pH 7.4], 150 mM NaCl, and 0.1% Tween-20) before blotting with primary antibodies against SRB1 (ab106572, 1:2000, Abcam; Shanghai, China) or β-actin (ab106572, 1:2000, Abcam) in blocking buffer at 4°C overnight, followed by incubation with secondary antibodies (ab151752, 1:2000, Abcam) for 60 min in the dark. Specific bands were visualized using an enhanced chemiluminescence detection kit.

### Immunofluorescence

Cells were fixed with 4% paraformaldehyde for 15 minutes, blocked with 5% serum (Solarbio) at 37°C for 60 minutes and incubated overnight at 4°C with a 1:300 dilution of anti‐glial fibrillary acidic protein (GFAP, ab7260, Abcam) antibodies. The cells were washed three times with PBS before treatment with a 1:100 dilution of a secondary antibody (Goat Anti-Mouse IgG, ab150117, Abcam) at 37°C for 2 hours. DAPI (4’,6-diamidino-2-phenylindole, Solarbio) was used for nuclear staining at 37°C for 2 minutes, and a Zeiss LSM 510 Meta Laser scanning confocal microscope was used to capture fluorescence images.

### Cell viability

Cell Counting Kit 8 (CCK-8) analysis (Beyotime; Shanghai, China) was used to assess cell viability. A total of 5,000 mAS cells underwent OGD/R treatment and/or transfection and were cultured in each well of a 96-well microplate. CCK-8 solution (10 μl) was added to the microplates and incubated for 2 h at 37°C. The absorbance at 450 nm was detected using a microplate reader (Bio–Rad).

### Lactate dehydrogenase assay

The death of mAS cells was measured using a cytotoxicity lactate dehydrogenase (LDH) assay kit (Dojindo; Shanghai, China) as directed by the manufacturer. Briefly, following OGD/R treatment and/or transfection, mAS cells were cultured in 6‐well plates. The mAS cells were collected, resuspended and cultured in 96‐well plates for specified times at 37°C in CO_2_. The cells were cultured for 30 minutes at 37°C in CO2 with 10 L of lysis buffer. Working solution (100 µL) was added to each well, and the samples were cultured at room temperature (RT) in the dark. After the addition of 50 µL stop solution to each well, LDH levels in the culture supernatant were measured using a microplate reader at 490 nm. The LDH levels of the control group are expressed as 100%, and the levels in the other groups were normalized to this value.

### Apoptosis

The Annexin V-FITC/PI Apoptosis Detection Kit (Sangon Biotech; Shanghai, China) was used to measure cell apoptosis. The mAS cells that underwent OGD/R treatment and/or transfection were seeded into 6-well plates (5 × 10^5^ cells/well) and reacted with 5 μl Annexin V-FITC and 10 μl PI in the dark at RT for 5 min. Flow cytometry analysis was performed in a FACS Verse flow cytometer (Becton Dickinson; San Jose) using FlowJo software (version 10; Treestar, Ashland, OR, USA).

### Measurement of ROS and superoxide dismutase (SOD) activities and malondialdehyde (MDA) content

Total ROS and SOD activities and MDA content in mAS cells were measured using an ROS Assay Kit (Beyotime), Total SOD Kit with NBT (Beyotime) and Lipid Peroxidation MDA Assay Kit (Beyotime), respectively, according to the manufacturer’s instructions.

### Enzyme-linked immunosorbent assay (ELISA)

The secretory levels of tumor necrosis factor-alpha (TNF-α), interleukin-1 beta (IL-1β), interleukin-6 (IL-6) and IL-10 (Beyotime; Shanghai, China) were measured using ELISA kits. OGD/R-treated and/or transfected cells were suspended and added to 96-well microplates for processing according to the manufacturer’s instructions. After 24 hours, the conditioned medium was collected from the wells. Absorbance at 450 nm was measured using a Bio–Rad Microplate Reader, and the amounts were estimated using a standard curve.

### Dual-luciferase reporter assay

The target sequences between circCTNNB1/SRB1 and miR-96-5p were predicted using StarBase (http://starbase.sysu.edu.cn/) online software. The 3′-UTR of circCTNNB1 or SRB1 containing miR‐96-5p binding sites was generated and subcloned into a pGL2‐Base vector (Promega; Madison, USA). The mAS cells were grown in 96‐well plates and co‐transfected using Lipofectamine™ 3000 with a combination of firefly luciferase reporter, pRL‐CMV Renilla luciferase reporter, and miR‐96-5p mimic or its mutant control. After 24 h, the relative luciferase activity was determined using a dual-luciferase reporter gene analysis system (Promega), and the Renilla luciferase reference plasmid was standardized.

### Mouse MCAO model

Male Kunming mice were used in this study. The animal committee at Kunming Medical University approved all animal experiment protocols. The procedures to establish the MCAO model were previously described [34022892]. Briefly, intraperitoneal anesthesia was administered to healthy adult Kunming mice (4% chloral hydrate). From a left lateral approach to the neck, the left common carotid artery, internal carotid artery, and external carotid artery were separated sequentially. A silicone cord was inserted from the common carotid artery to the middle cerebral artery. The silicone cord was removed and ligated after 60 minutes of embolization. Sham-operated mice underwent the same surgery as the experimental mice, except that the middle cerebral artery was not occluded.

Mice were randomly divided into five groups: (a) Control group (n = 5); (b) Sham group (n = 5); (c) MCAO group (n = 5); (d) MCAO + OE-circCTNNB1: mice were administered OE-circCTNNB1 (0.5 mg·kg^−1^, via intracerebroventricular injection) before ischemia treatment (n = 5) and (e) MCAO + OE-circCTNNB1 + si-SRB1: mice were administered OE-circCTNNB1 and si-SRB1 (0.5 mg·kg^−1^, via intracerebroventricular injection) before ischemic treatment (n = 5). OE-circCTNNB1 and/or si-SRB1 were injected into the left cerebral ventricle of mice. The MCAO surgery was performed one day after the injection.

### Neurobehavioral tests

Neurobehavioral tests were performed on mice (n = 5 per group) to assess functional results. Prior to MCAO, the animals were trained, and impairments were measured 24 hours later. The neurological severity score (NSS) [26] ranges from 0 to 4: 0, normal mouse; 1, mouse failed to extend left forepaw; 2, mouse circled to left; 3, mouse fell to the left; and 4, no spontaneous movement.

### Measurement of infarct volume

The brain was removed and cut into 5 pieces using a brain matrix after MCAO for 24 hours. The sections were immersed in pre-heated 2% 2,3,5-triphenyltetrazole chloride (Aladdin; Shanghai, China) in saline for 30 minutes then fixed in 4% paraformaldehyde overnight. A blind observer evaluated the infarct volume using Image-Pro Plus 5.1 software. The actual infarct volume with edema correction was calculated by subtracting the non-infarct volume of the ipsilateral hemisphere from the volume of the contralateral hemisphere.

### Assessment of BBB integrity

Three hours before sacrifice, 2% Evans blue (Solarbio), dissolved in 0.9% saline, was injected intravenously. After 2 h of circulation, mice from each group were administered 4% chloral hydrate anesthesia (50 mg/kg) through the left ventricle to remove the intravascular cavity. Evans blue dye continued until the fluid from the right atrium became colorless. Mice were beheaded, and the brains were extracted swiftly. The brains were weighed and homogenized in 4 ml of a 50% formamide solution. Following centrifugation, the concentration of Evans blue was measured using a spectrophotometer at 620 nm for absorbance against a standard curve. Evans blue content in brain tissue (μg/g) = A × A phthalein amine amount, according to the standard curve established by the Venturi blue content:

### Assessment of brain water content

The mice were sacrificed, and their brains were isolated shortly after anesthetization with 4% chloral hydrate. The brains were weighed using a precise electronic balance (wet weight) and dried for 24 h at 110°C before being weighed again (dry weight). Brain water content (%) = (wet weight – dry weight)/wet weight×100%.

### Terminal deoxyribonucleotide transferase-mediated nick end labeling (TUNEL) assay

To detect apoptotic cells in the mouse brain, a TUNEL test was performed using a One‐Step TUNEL Apoptosis Assay kit (Roche; Basel, Switzerland). Four‐micrometer‐thick paraffin slices were deparaffinized, hydrated, treated with proteinase K for 20 minutes and incubated in a humidified environment at 37°C for 1 hour with a combination of a fluorescently labeled solution of dUTP and the terminal deoxynucleotidyl transferase enzyme. The positive control was incubated with DNase I at RT for 10 minutes before the fluorescent labeling procedure. Negative controls were incubated with dUTP at RT for 10 minutes. Following DAPI staining, the apoptotic cells were measured using a Nikon Eclipse 80i microscope (Nikon Corporation).

### Statistical analysis

GraphPad Prism 8.0 (GraphPad Software; San Diego, USA) was used for statistical analyses. Data are expressed as the means ± standard deviation. Student’s *t*-test was used to analyze the two groups. Three or more treatments or groups were compared using a one-way analysis of variance followed by Tukey’s test. *P* < 0.05 was considered a significant difference.

## Results

To examine the functions of circCTNNB1 in cerebral IRI, we used RT-qPCR to examine circCTNNB1 expression in OGD/R-treated mAS. The biological function of circCTNNB1 overexpression in cellular apoptosis, oxidative stress and inflammation was detected. We also performed a dual-luciferase reporter assay to determine whether circCTNNB1 sponging of miR-96-5p regulated SRB1 expression in mAS. The results are described below.

### CircCTNNB1 is expressed at low levels after OGD/R treatment

**Figure 1.**
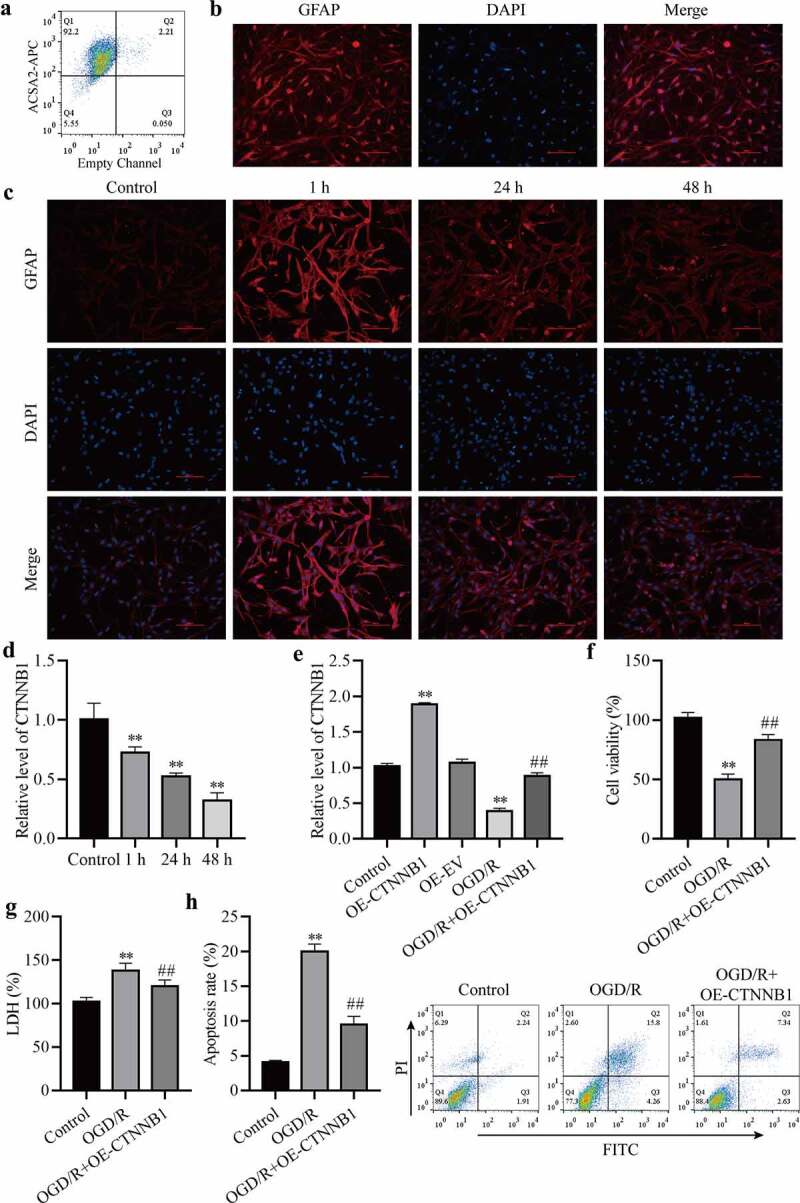
circCTNNB1 expression was low after OGD/R treatment. (a) Flow cytometry analysis of ACSA2-APC in ACSA2+ cells. (b) Immunofluorescence confirmed that purified astrocytes expressed GFAP. (c) Immunofluorescence determined the mAS cell activation state. Scale bar = 100 cm. (d and e) RT-qPCR assessed circCTNNB1 expression. (f) CCK-8 was used to measure mAS cell activity. (g) The death of mAS cells was measured using LDH. (h) Cell apoptosis was measured using an FITC/PI kit. **P < 0.01 vs. Control; ^##^P < 0.01 vs. OGD/R.

To detect activation changes in mAS cells after OGD/R treatment. We purified mAS cells from the cerebral cortex. As shown in Figure 1(a), XXX% of the microbead-labeled cells were ACSA2 + . Immunofluorescence staining confirmed that over 90% of bead-bonded cells expressed GFAP (Figure 1(b)). The activation of mAS cells was elevated 1 h after OGD/R compared to the control but reduced after OGD/R at 24 h and 48 h compared to 1 h (Figure 1(c)). RT-qPCR detected circCTNNB1 expression levels. OGD/R decreased circCTNNB1 levels over time compared to the control group (Figure 1(d)). To detect the effect of circCTNNB1 on OGD/R, we overexpressed circCTNNB1 in mAS cells. The transfection efficiency of OE-circCTNNB1 was verified using RT‐qPCR (Figure 1(e)). The functions of circCTNNB1 on OGD/R-induced mAS cells were estimated using cell viability, apoptosis and LDH content. Overexpression of circCTNNB1 significantly increased the viability of mAS cells compare to the OGD/R group (figure 1(f)). Cell apoptosis and LDH contents also increased after OGD/R treatment, and the upregulation of circCTNNB1 inhibited OGD/R‐induced apoptosis and LDH release (Figure 1(g) and (h)). These results indicated that OGD/R induced cell injury by downregulating circCTNNB1 levels.

### circCTNNB1 is involved in oxidative stress and inflammation after OGD/R treatment

**Figure 2.**
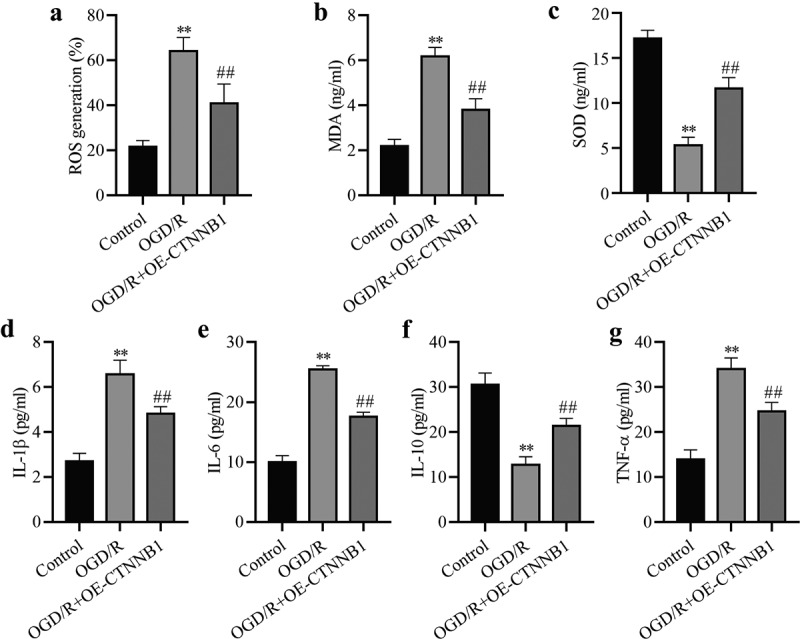
circCTNNB1 is involved in oxidative stress and inflammation after OGD/R treatment. The ROS (a) and SOD (b) activities and MDA (c) content in mAS cells were measured using an ROS Assay Kit, Total SOD Kit and Lipid Peroxidation MDA Assay Kit, respectively. ELISA measured IL-1β (d), IL-6 (e), IL-10 (f) and TNF-α (g) levels. **P < 0.01 vs. Control; ^##^P < 0.01 vs. OGD/R.

To understand the effects of circCTNNB1 on oxidative stress, the levels of ROS, MDA and SOD were measured in mAS cells after OGD/R treatment using commercial kits. OGD/R increased the ROS and MDA levels. Overexpression of circCTNNB1 partially reduced ROS and MDA levels (Figure 2(a) and (b)). The overexpression of circCTNNB1 increased the levels of SOD in OGD/R-treated mAS cells. T detect the roles of circCTNNB1 in the inflammatory response, TNF-α, IL-1β, IL-6 and IL-10 levels were measured using ELISA. OGD/R treatment increased the levels of TNF-α, IL-1β, and IL-6 in mAS cells, and the levels of IL-10 decreased. However, overexpression of circCTNNB1 decreased TNF-α, IL-1β, and IL-6 levels and increased IL-10 levels (Figure 2(d–f) and (g)). These data suggested that circCTNNB1 negatively regulated oxidative stress and the inflammatory response in OGD/R-treated mAS cells.

### Increasing circCTNNB1 attenuates OGD/R-induced injury by up-regulating SRB1 expression

**Figure 3.**
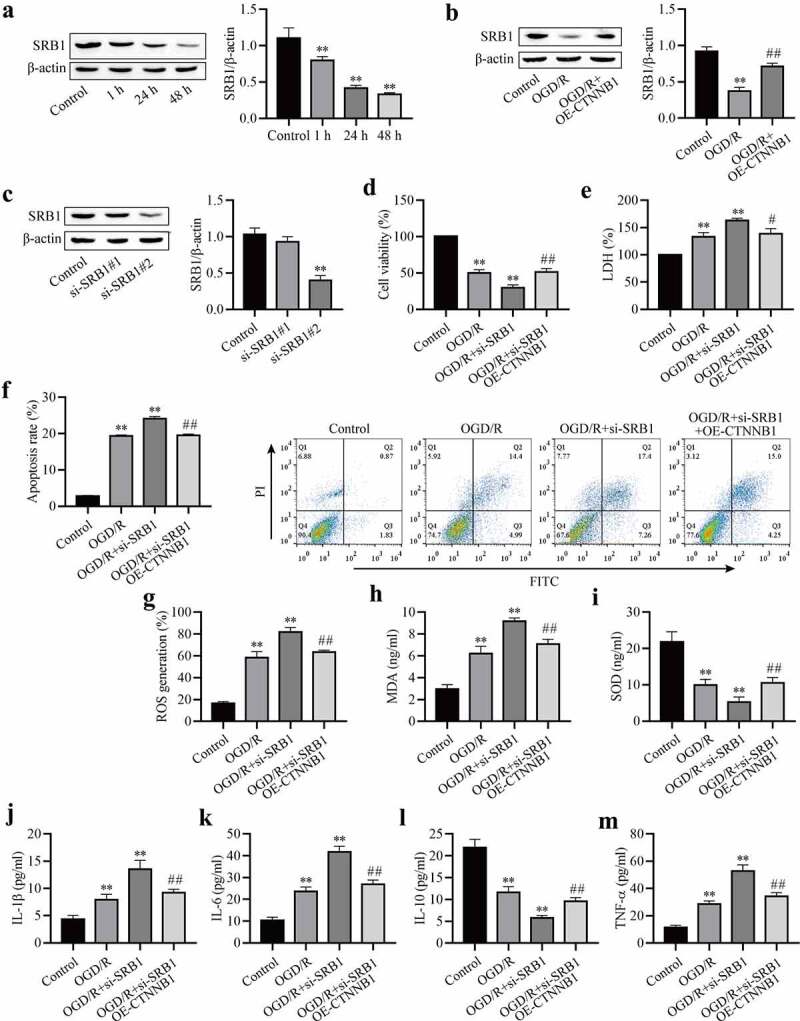
CircCTNNB1 attenuates OGD/R injury by upregulating SRB1 expression. (a-c) Western blotting measured SRB1 protein expression. (d) CCK-8 was used to measure mAS cell activity. (e) The death of mAS cells was measured using LDH. (f) Cell apoptosis was measured using an FITC/PI kit. The ROS (g) and SOD (h) activities and MDA (i) content in mAS cells were measured using an ROS Assay Kit, Total SOD Kit and Lipid Peroxidation MDA Assay Kit, respectively. ELISA measured IL-1β (j), IL-6 (k), IL-10 (l) and TNF-α (m) levels. **P < 0.01 vs. Control; ^#^P < 0.05 and ^##^P < 0.01 vs. si-SRB1.

SRB1 is a physiologically relevant receptor for HDL. The expression level of SRB1 was measured in OGD/R-induced mAS cells. The expression of SRB1 in OGD/R-induced mAS cells was downregulated with increasing reoxygenation time (Figure 3(a)), which suggests that SRB1 plays an important role in OGD/R injury. We hypothesized that circCTNNB1 exerted its function by regulating SRB1 expression. The effect of circCTNNB1 on SRB1 was examined using Western blotting. The overexpression of circCTNNB1 partially rescued OGD/R-inhibited SRB1 expression (Figure 3(b)). To confirm whether SRB1 mediated the injury caused by circCTNNB1 in OGD/R-induced mAS cells, mAS cells were transfected with si-SRB1 and/or OE-circCTNNB1 and treated with OGD/R. The transfection efficiency of si-SRB1 was identified using Western blotting (Figure 3(c)). Si-SRB1#1 did not reduce SRB1 expression, but si-SRB1#2 significantly reduced SRB1 expression. Si-SRB1#2 achieved more effective knockdown efficiency. Therefore, si-SRB1#2 was used for subsequent experiments. Knockdown of SRB1 further aggravated the OGD/R-induced damage to mAS cells, but transfection with si-SRB1 and overexpression of circCTNNB1 partially alleviated the cell survival, oxidative damage and inflammation induced by OGD/R (Figure 3(d-m)). These data suggest that circCTNNB1 plays an important role in OGD/R injury by regulating SRB1 expression.

### circCTNNB1 regulates SRB1 protein expression by sponging miR-96-5p

**Figure 4.**
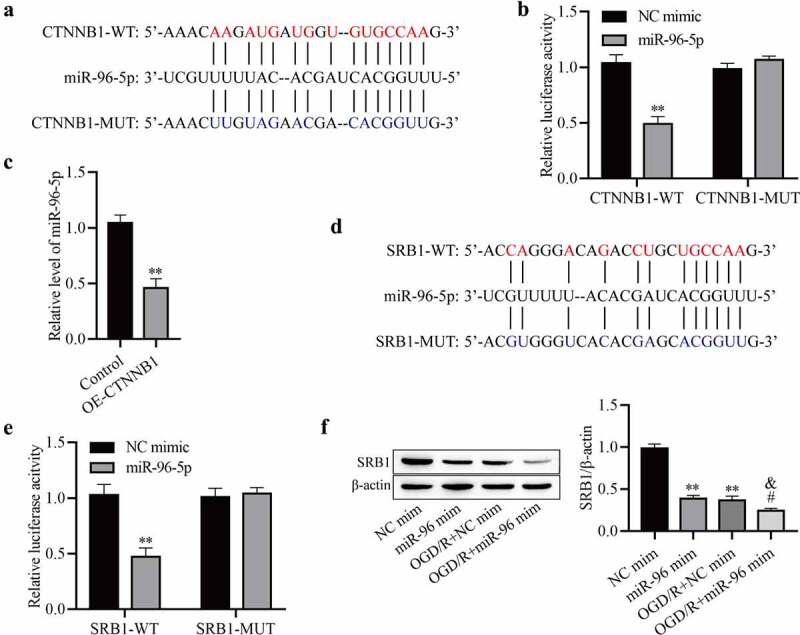
CircCTNNB1 regulates SRB1 protein expression by sponging miR-96-5p. (a) The binding sites between miR-96-5p and WT or MUT circCTNNB1. (b) Luciferase activity was determined using a dual-luciferase assay. (c) RT-qPCR assessed miR-96-5p expression. (d) The binding sites between miR-96-5p and WT or MUT SRB1. (e) Luciferase activity was determined using dual-luciferase assays. (f) Western blotting measured SRB1 protein expression. **P < 0.01 vs. Control; ^#^P < 0.05 vs. miR-96-5p mimic; ^&^P < 0.05 vs. OGD/R+ miR-NC mimic.

MiR‐96-5p bound to complementary sequences in circCTNNB1 (Figure 4(a)). MiR-96-5p was related to cerebral ischemia injury in rats, but the function of miR-96-5p in cerebral IRI was not clear [27]. To further ascertain whether miR-96-5p bound to circCTNNB1, we performed a luciferase reporter assay. Luciferase activity was obviously downregulated in mAS cells co‐transfected with circCTNNB1-WT and miR‐96-5p mimic, but it did not change after transfection with circCTNNB1-MUT (Figure 4(b)). After up-regulation of circCTNNB1, the miR-95-5p level was significantly decreased (Figure 4(c)). These results indicated that circCTNNB1 negatively regulated miR-95-5p in mAS cells. We also screened the target sites of miR‐95‐5p and SRB1 using StarBase (Figure 4(d)). SRB1‐WT decreased luciferase activity in the miR‐95‐5p mimic group but did not affect luciferase activity of the NC mimic group (Figure 4(e)). OGD/R treatment significantly reduced SRB1 expression, but the decrease was reversed by miR‐96-5p mimic treatment (figure 4(f)). Taken together, these results suggest that circCTNNB1 regulates SRB1 expression by sponging miR-96-5p.

### *miR-96-5p overexpression reverses the protective effect of OE-circCTNNB1 against OGD/R‐induced injury* in vitro

**Figure 5.**
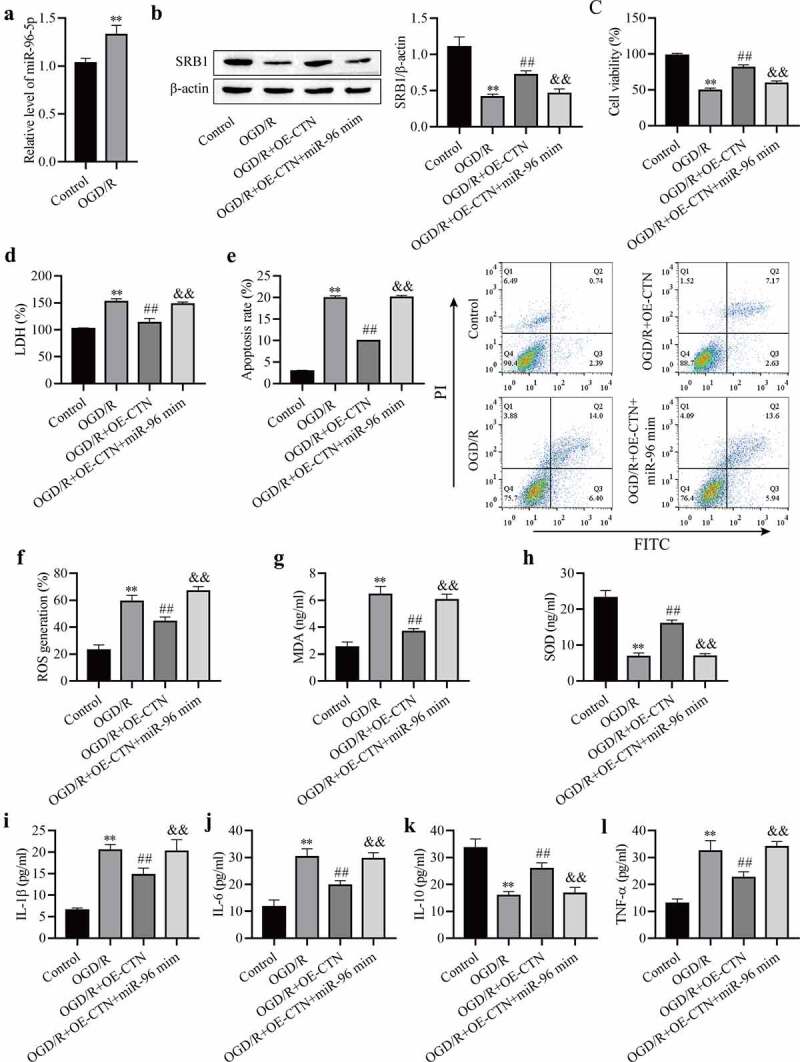
MiR-96-5p reverses the protective effect of circCTNNB1 upregulation against OGD/R‐induced injury *in vitro*. (a) RT-qPCR assessed miR-96-5p expression. (b) Western blotting measured SRB1 protein expression. (c) CCK-8 was used to measure mAS cell activity. (d) The death of mAS cells was measured using LDH. (e) Cell apoptosis was measured using an FITC/PI kit. The ROS (f) and SOD (g) activities and MDA (h) content in mAS cells were measured using an ROS Assay Kit, Total SOD Kit and Lipid Peroxidation MDA Assay Kit, respectively. ELISA measured IL-1β (i), IL-6 (j), IL-10 (k) and TNF-α (l) levels. **P < 0.01 vs. Control; ^##^P < 0.01 vs. OGD/R; ^&&^P < 0.01 vs. OGD/R+ OE-CTNNB1.

OGD/R increased miR-96-5p expression in mAS cells (Figure 5(a)), and miR-96-5p may be involved in cerebral IRI. The mAS cells were pretreated with the miR-96-5p mimic, transfected with OE-circCTNNB1, and exposed to OGD/R. SRB1 expression was measured using Western blotting, and the results showed that up-regulated circCTNNB1 partially rescued OGD/R-inhibited SRB1 protein levels, and transfection of the miR-96-5p mimic plus OE-circCTNNB1 inhibited SRB1 (Figure 5(b)). These results further demonstrated that circCTNNB1 sponged miR-96-5p to regulate SRB1 protein expression. Transfection of OE-circCTNNB1 alleviated the OGD/R-induced decrease in cell viability and LDH leakage and increased apoptosis, but these effects of circCTNNB1 were abolished by transfection of the miR-96-5p mimic plus OE-circCTNNB1 (Figure 5(c,e)). Transfection of the miR-96-5p mimic with OE-circCTNNB1 simultaneously abolished the protective activities of OE-circCTNNB1 against OGD/R-induced oxidative stress and the inflammatory response (figure 5(f-l)). These data suggest that circCTNNB1 protects against OGD/R by downregulating miR-96-5p levels.

### *circCTNNB1 and SRB1 are involved in cerebral IRI* in vivo

**Figure 6.**
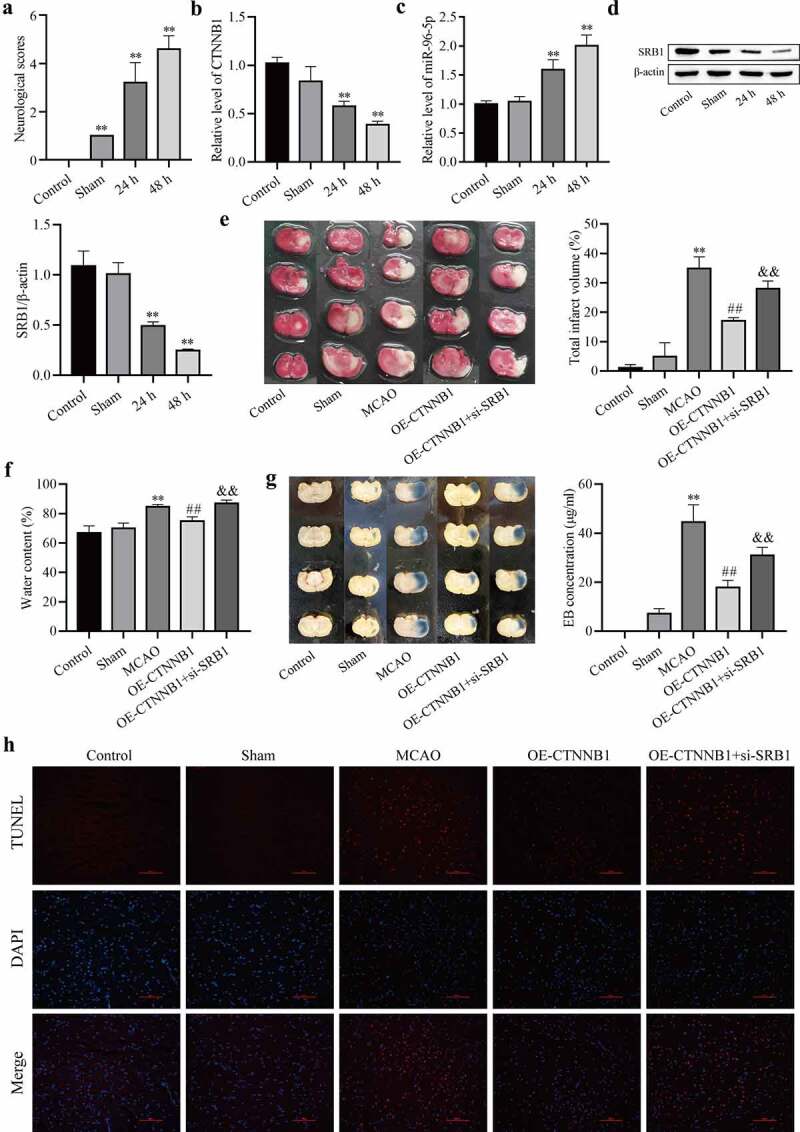
CircCTNNB1 and SRB1 are involved in cerebral IRI *in vivo*. (a) Neurological severity score. (b and c) RT-qPCR assessed the expression of circCTNNB1 and miR-96-5p. (d) Western blotting measured SRB1 protein expression. (e) 2,3,5-Triphenyltetrazole chloride staining. (f) Water content. (g) Evans blue staining. (h) TUNEL revealed cell apoptosis in MCAO mouse brains. Scale bar = 100 Dm. **P < 0.01 vs. Control; ^##^P < 0.01 vs. MCAO; ^&&^P < 0.01 vs. OE-CTNNB1.

To demonstrate the protective role of circCTNNB1 and SRB1 against cerebral IRI, an MCAO mouse model was established. Neurobehavioral tests were performed, and NSS was increased after MCAO (Figure 6(a)). These results indicated that the MCAO model was established. RT-qPCR and Western blotting were used to detect the expression levels of circCTNNB1, miR-96-5p, and SRB1. As shown in Figure 6(b-d), circCTNNB1 and SRB1 levels were down-regulated after MCAO, and miR-96-5p levels were up-regulated. We determined the effects of circCTNNB1 and SRB1 on infarction, BBB integrity and brain water content. The infarct regions, BBB integrity and brain water content were increased in the MCAO groups. Up-regulation of circCTNNB1 significantly reduced brain injury, but brain injury was partially aggravated in the MCAO group and transfection of circCTNNB1 plus si-SRB1 (Figure 6(e-g)). TUNEL analysis revealed that up-regulation of circCTNNB1 ameliorated cell apoptosis in MCAO mouse brains, but the effect was abolished by inhibition of SRB1 (Figure 6(h)). These data indicate that circCTNNB1 and SRB1 play protective roles in cerebral IRI.

## Discussion

Ischemic stroke or cerebral ischemia has overtaken heart disease as the leading cause of death and morbidity globally [28,29]. Oxidative stress and inflammation are pronounced phenomena in IRI [30]. Therefore, regulating oxidative stress and inflammation is vital for cerebral IRI [31,32]. The BBB, which is composed of endothelial, astrocytes, and microglial cells, is responsible for the entry of inflammatory cells and influences the degree of brain injury and recovery [33]. According to one report, the regulation of astrocyte function is part of maintaining BBB integrity and has promise in the treatment of cerebral IRI [34]. The current study found that circCTNNB1 ameliorated OGD/R-induced cell apoptosis, oxidative stress damage and the inflammatory response by sponging miR-96-5p to up-regulate SRB1 expression. The up-regulation of circCTNNB1 and SRB1 alleviated brain damage in a mouse MCAO model.

Although evidence demonstrated a protective role of CTNNB1 in cerebral IRI [35,36], the expression and biological role of circCTNNB1, a highly homologous gene of CTNNB1, in cerebral IRI was not characterized. The present study confirmed the down-regulation of circCTNNB1 after OGD/R treatment. Reduced circCTNNB1 expression was also found in the MCAO mouse model. Up-regulation of circCTNNB1 alleviated the OGD/R-inhibited cell viability of astrocytes and IRI-induced damage to the brain. The present study found that overexpression of circCTNNB1 reversed the effects of OGD/R treatment, increased the levels of TNF-α, IL-1β, and IL-6 in mAS cells, and decreased the levels of IL-10. Astrocytes have powerful pro-inflammatory potential in the neuroinflammatory response [37], which suggests that OGD/R promoted the inflammatory response by increasing mAS injury, but overexpression of circCTNNB1 inhibited the inflammatory response by ameliorating OGD/R-induced mAS injury. Taken together, these results demonstrated that cicrCTNNB1 was critical for cerebral IRI and functioned as a negative regulator in cerebral IRI progression.

CircRNAs exhibit widespread functions in multiple biological processes. CircRNAs are potential biomarkers for cancer, cardiovascular disease and cerebral ischemic stroke [38–40]. Several recent studies suggested that circRNAs may also be biomarkers for neurological disorders. CircRNA cZHF292 silencing alleviated rat neural stem cell injury after OGD/R [41]. CircRNA HECTD1 is involved in cerebral ischemic stroke by affecting astrocyte autophagy [42]. The present study indicated that circCTNNB1 may be a potential biomarker for the diagnosis and treatment of cerebral IRI.

CircRNAs may function as endogenous sponges that interact with miRNAs to control the expression of miRNA target genes. Our study revealed that circCTNNB1 acted as an miR-96-5p sponge to regulate astrocyte survival. MiR-96-5p plays pleiotropic roles in cancer [43], wound healing [44], placental dysfunction [45] and hepatic steatosis [46]. Consistent with prior results, the miR-96-5p expression level was elevated following cerebral ischemia [27]. To the best of our knowledge, the current study is the first study to investigate the specific function of miR-96-5p in cerebral IRI. Our study also found that miR-96-5p was up-regulated in OGD/R-induced astrocytes. Astrocytes were co-transfected with OE-circCTNNB1 and miR-96-5p mimic to determine whether circCTNNB1-mediated functional effects were unique to miR-96-5p. Up-regulation of miR-96-5p remarkably counteracted the protective effect of OE-circCTNNB1 on astrocyte abilities. These results revealed that cricCTNNB1 was involved in cerebral IRI by regulating miR-96-5p.

SRB1 is an integral membrane protein found mostly in the liver and endocrine organs that make steroids and functions as an HDL receptor [18]. Increasing evidence suggests that SRB1 plays important roles in cardiovascular disease and neurological disorders. Hepatic overexpression of SRB1 in mice increased hepatocellular cholesterol absorption and bile secretion, decreased circulating HDL-C levels, and reduced atherosclerosis [47]. SRB1 plays a role in C1q-facilitated β-amyloid clearance by astrocytes in Alzheimer’s disease [48]. SRB1 mediates the cellular uptake of vitamin E. Mouse embryos lacking SRB1 are vitamin E deficient, and approximately half of these embryos fail to seal the neural tube, which results in cephalic neural tube abnormalities [49]. However, there is no evidence indicating that SRB1 is involved in the protection of the BBB after cerebral IRI. The current study identified the regulatory mechanism of SRB1 in the pathogenesis of cerebral IRI. SRB1 was decreased in OGD/R injury mAS cells and MCAO mice. SRB1 silencing aggravated OGD/R-induced astrocyte damage and mouse brain injury after IRI. However, up-regulation of circCTNNB1 partially alleviated the damage. We also considered that circCTNNB1 acted as an miR-96-5p sponge to regulate SRB1 expression. This study is the first study to show that SRB1, as a downstream effector of circCTNNB1, controlled astrocyte survival after OGD/R. Therefore, we speculate that the circCTNNB1-miR-96-5p-SRB1 axis contributes to the protection of astrocytes after cerebral IRI. However, the detailed mechanism by which SRB1 facilitates viability in OGD/R-treated astrocytes, especially the process involved in regulating oxidative stress and inflammation, requires further study.

## Conclusions

Our findings revealed previously unknown circCTNNB1 regulatory mechanisms that protected astrocytes from OGD/R-induced oxidative stress and inflammation by modulating miR-96-5p/SRB1. These findings support circCTNNB1 and SRB1 as new targets for the treatment of cerebral IRI.

## Research highlights

CircCTNNB1 and SRB1 were significantly down-regulated and miR-96-5p was overexpressed in cerebral IRI.

CircCTNNB1 sponged miR-96-5p to regulate SRB1 expression in mAS.

Overexpression of circCTNNB1 ameliorated cerebral IRI via regulation of the miR-96-5p/SRB1 axis.
